# Relationship between heart rate variability and cognitive function in patients with enlarged perivascular space

**DOI:** 10.3389/fnagi.2022.1031031

**Published:** 2022-11-07

**Authors:** Dongyang Zhou, Chang Lu, Chunhe Su, Yuechen Liu, Jing Chen, Feng Zhang, Hongying Bai, Qianqian Li

**Affiliations:** ^1^Department of Neurology, The Second Affiliated Hospital of Zhengzhou University, Zhengzhou, China; ^2^Department of Neurology, Huiji District People’s Hospital, Zhengzhou, China

**Keywords:** enlarged perivascular space, cognitive impairment, basal ganglia, heart rate variability, cerebral small vessel disease, autonomic nervous system

## Abstract

**Objective:**

To explore the relationship between heart rate variability (HRV), the brain distribution of enlarged perivascular space (EPVS), and cognitive impairment in patients with EPVS.

**Materials and methods:**

The clinical and imaging data of 199 patients with EPVS were retrospectively analyzed. EPVS load in the basal ganglia (BG) and centrum semiovale (CS) regions were assessed using the Potter’s method. Cognitive function was evaluated using the Montreal Cognitive Assessment Scale. A logistic regression model was used to analyze the relationship between HRV, the brain distribution of EPVS and cognitive function in patients with EPVS. A receiver operating characteristic curve was used to assess the predictive value of HRV for cognitive function in patients with EPVS.

**Results:**

Of the 199 patients, 27 and 42 presented with severe BG-EPVS and cognitive impairment, respectively. Significant differences were observed in the root mean square of successive differences of normal-normal (NN) intervals for period of interest (rMSSD), the percentage of adjacent NN intervals greater than 50 ms (PNN50), and the ratio of low-frequency power (LF) to high-frequency power (HF) between the mild and severe BG-EPVS groups (*P* < 0.05). Patients who presented with and without cognitive impairment differed significantly in the standard deviation of NN intervals (SDNN), rMSSD, PNN50, total power, LF, and LF/HF (*P* < 0.05). rMSSD (odds ratio [OR] 0.871, 95% confidence interval [CI] 0.768–0.988) and LF/HF (OR 3.854, 95% CI 1.196–12.419) were independent influencing factors of BG-EPVS, and rMSSD (OR 0.936, 95% CI 0.898–0.976) was an independent influencing factor of cognitive impairment in patients with EPVS. The optimal cut-off point was 0.312, with an area under the curve of 0.795 (95% CI 0.719–0.872) for predicting cognitive impairment in patients with EPVS by rMSSD.

**Conclusion:**

Reduced HRV is involved in the pathophysiological mechanisms of the formation and development of BG-EPVS and is associated with cognitive impairment in patients with EPVS, independent of CS-EPVS. For patients with HRV changes but without autonomic nervous system symptoms, positive intervention may slow the occurrence or progression of EPVS and cognitive impairment in patients with EPVS.

## Introduction

With the increasing aging process and the development of magnetic resonance imaging (MRI), enlarged perivascular space (EPVS) is observed more frequently (Wardlaw et al., 2013a) and the harmful effects of EPVS have garnered growing attention. The perivascular space is an interstitial fluid-filled cavity surrounding the penetrating small arteries and veins. It is a critical drainage channel for interstitial fluid and solutes in the brain and may be enlarged for various physiological and/or pathological reasons (Caunca et al., 2019). EPVS has recently been identified as an early imaging manifestation of cerebral small vessel disease (CSVD) (Wardlaw et al., 2013b). It often appears in the basal ganglia (BG) and centrum semiovale (CS) and can also be observed in the hippocampus and midbrain (Chen et al., 2019). Reportedly, EPVS is not only associated with vascular dementia, but also with increased risk or severity of other types of dementia (Smeijer et al., 2019; Jie et al., 2020; Paradise et al., 2021). Conversely, factors that influence EPVS cognitive impairment and the underlying mechanisms are not fully understood.

Heart rate variability (HRV) has become an important and recognized non-invasive tool for evaluating autonomic nervous system (ANS) function. According to previous studies, the decline in HRV is associated with the progression of white matter hyperintensity (WMH) (Moon et al., 2018; Lee et al., 2021; Tian et al., 2021), suggesting that ANS disorders are closely associated with the development of CSVD. However, the relationship between HRV and EPVS, which is a main marker of CSVD, is still unclear. Moreover, a study has shown that a decline in HRV is associated with poorer cognitive performance (Forte et al., 2019). Similarly, research on the relationship between cognitive impairment and HRV in patients with EPVS is more limited.

Therefore, we hypothesized that the reduction in HRV is not only related to the occurrence and development of EPVS, but may also play a crucial role in the development of cognitive impairment in patients with EPVS. This study aimed to further reveal the pathogenesis of EPVS and discuss the possibility of HRV as a basis for early diagnosis, identification, and intervention of EPVS-related cognitive impairment. We also aimed to determine new clinical ideas and provide data that may serve as a reference for the early prevention, diagnosis, and treatment of cognitive impairment in patients with EPVS.

## Materials and methods

### Patients

**FIGURE 1:**
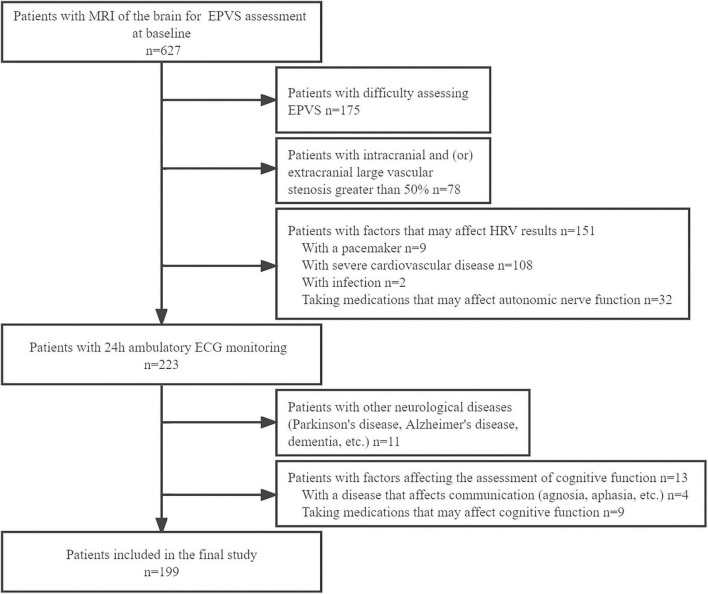
Flowchart of enrollment of study patients. MRI, magnetic resonance imaging; EPVS, enlarged perivascular spaces; HRV, heart rate variability; ECG, electrocardiogram.

This retrospective study involved patients who had been hospitalized in the Department of Neurology of the Second Affiliated Hospital of Zhengzhou University between January 2020 and May 2022 (Figure 1). The inclusion criteria were: (1) patients aged ≥35 years, (2) patients having undergone a 3.0T cranial MRI scan, and (3) patients or their authorized persons having provided signed informed consent. The exclusion criteria were: (1) patients whose EPVS could not be easily assessed; (2) vascular examination findings suggestive of intracranial and/or extracranial large vessel stenosis >50%; (3) presence of a pacemaker and/or severe cardiovascular diseases such as cardiomyopathy, arrhythmias, and other conditions that might have affected the HRV; (4) infection; (5) intake of medication, such as beta-blockers or calcium antagonists, which might have affected the ANS function prior to HRV monitoring; (6) presence of other neurological disorders, such as Parkinson’s disease, Alzheimer’s disease, or dementia; (7) presence of aphasia and other disorders that might have affected effective communication; and (8) use of drugs that could have affected cognition, such as donepezil, in the preceding 3 months the start of the study.

### Data collection

Baseline patient information was collected, including sex, age, smoking history (former smoker and quit <3 months prior, or current smoker of >1 cigarette per day), history of alcohol consumption (average daily alcohol intake ≥25 g for >1 year, quit <3 months prior), and history of underlying diseases, such as hypertension and diabetes mellitus. Laboratory findings included total cholesterol, low-density lipoprotein cholesterol, triglyceride, high-density lipoprotein cholesterol, and homocysteine.

### Heart rate variability monitoring

Participants were monitored with a 12-lead dynamic ECG recorder using DMS300-4A (Diem Software Limited, Beijing, China) for long-range HRV (>18 h). HRV data were collected and reviewed by a specialist electrocardiogram (ECG) physician who was blinded to the patient’s MRI and clinical data. The HRV frequency domain parameters were as follows: total power (TP), the high- (HF) and low-frequency (LF) power, and ratio of LF to HF (LF/HF). The time domain parameters were the standard deviation of normal-normal (NN) intervals (SDNN), the root mean square of successive differences of NN intervals for period of interest (rMSSD), the standard deviation of the mean of NN intervals every 5 min for the period of interest (SDANN), the percentage of adjacent NN intervals greater than 50 ms (PNN50), and the mean of NN intervals standard deviation every 5 min for period of interest (SDNNindex).

### Imaging

Head MRI (Skyra3.0T, Siemens, Munich, Germany) was obtained within 1 week of admission. The sequences included T1-weighted, T2-weighted, fluid attenuated inversion recovery imaging (FLAIR), susceptibility-weighted imaging, and diffusion-weighted imaging. The severity assessment of EPVS in different areas of the brain was performed separately by two neurologists, and disagreements were resolved *via* a consensus following discussion.

**FIGURE 2:**
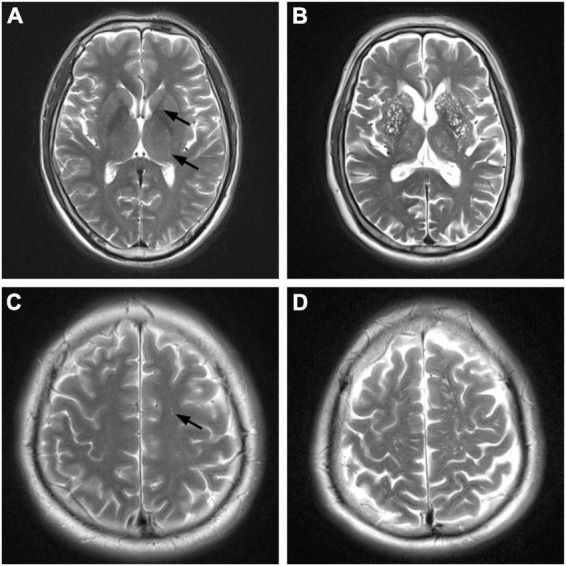
Magnetic resonance imaging (MRI) manifestations of different degrees of enlarged perivascular space (EPVS) (T2-weighted imaging). BG, basl ganglia; CS, centrum semiovale. **(A)** Mild BG-EPVS. **(B)** Severe BG-EPVS. **(C)** Mild CS-EPVS. **(D)** Severe CS-EPVS.

Enlarged perivascular space was defined as a lesion with clear boundaries, with signal intensity like that of the cerebrospinal fluid in all sequences. No high enhancement edge was observed on the FLAIR sequence. They were round or linear, and <3 mm in diameter. Potter et al.’s (2015) visual semi-quantitative assessment method was used to assess the BG-EPVS and CS-EPVS, with EPVS scores of 1 (1–10), 2 (11–20), 3 (21–40), and 4 (>40). The BG-EPVS patients were divided into two groups, mild and severe, with scores of 1–2 and 3–4, respectively. Similarly, the CS-EPVS patients were divided into two groups, mild and severe, with scores of 1–2 and 3–4, respectively (Figure 2).

White matter hyperintensity was defined as irregular abnormal signal in the white matter region of the brain, with high signal on T2-weighted imaging or FLAIR sequences, and its severity was assessed using the Fazekas score (Fazekas et al., 1987), with a Fazekas score ≥3 being considered severe.

Lacune was defined as a round or ovoid cavity measuring 3–15 mm with a low signal on FLAIR imaging, surrounded by a high-signal shadow, a low signal on T1-weighted imaging, and a high signal on T2-weighted imaging.

Cerebral microbleeds (CMBs) were defined as small, homogeneous round or oval foci of signal on susceptibility-weighted imaging of 2–5 mm in diameter.

### Cognitive function assessment

Two neuropsychologically trained neurologists with 6 years clinical experience evaluated the cognitive function of the patients using a uniform and standardized guideline. In case of inconsistent results, the supervising physician was asked to discuss the disputed results to determine the final score. The Beijing version of the Montreal Cognitive Assessment Scale was used to assess cognitive function, and the procedures were explained to the patients before the test to obtain good cooperation.

To correct for educational bias, one point was added for those with less than 12 years of education. A total score of ≥26 denoted no cognitive impairment, whereas a total score of <26 denoted cognitive impairment.

### Statistical analysis

Categorical variables were expressed as percentages, and comparisons between groups are made using Pearson’s chi-square or Fisher’s exact test. Continuous, normally distributed data were expressed as means and standard deviations, and non-parametric data were described as medians and interquartile ranges. The two independent samples *t*-test or Mann–Whitney *U*-test was used for comparison between groups of continuous variables. The information on statistically significant HRV indicators between the groups was subjected to logistic regression analysis to analyze independent influences. The HRV metric was used to build a predictive model of cognitive impairment in patients with EPVS, plot the receiver operating characteristic curve, identify the optimal cut-off point, and calculate the area under the curve. Statistical analyses were performed using the Statistical Product Service Solutions (SPSS) software version 26.0 (International Business Machines Corporation (IBM) Corp., Armonk, NY, USA). The significance level was set at *P* < 0.05 (two-tailed).

## Results

### Statistical description

Our study included 199 patients, 105 (52.8%) men and 94 (47.2%) women, with a mean age of 63.2 ± 11.6 years, grouped by degree of EPVS: 27 cases in the severe BG-EPVS group and 23 in the severe CS-EPVS group. According to the Montreal Cognitive Assessment Scale score: 157 cases were without cognitive impairment, whereas 42 had cognitive impairment.

### Influencing factors of enlarged perivascular space in basal ganglia and centrum semiovale

A statistically significant difference was observed between the mild and severe BG-EPVS groups in terms of sex, age, presence of severe paraventricular WMH (sPWMH), presence of severe deep WMH (sDWMH), presence of lacune, rMSSD, PNN50, and LF/HF (*P* < 0.05). Compared to the mild BG-EPVS group, the severe BG-EPVS group exhibited a trend of comprising more men, older individuals, higher LF/HF, and increased sPWMH, sDWMH, and lacune. There was a trend toward lower rMSSD and PNN50 in the severe BG-EPVS group than those in the mild group, and the difference was statistically significant (*P* < 0.05). For the CS-EPVS group, no statistically significant differences were found between the mild and severe CS-EPVS subgroup data (*P* > 0.05) (Table 1).

**TABLE 1:** Univariate analysis of factors related to enlarged perivascular space (EPVS) load in different brain regions.

Characteristics	BG-EPVS		Test statistic	*P*-value	CS-EPVS		Test statistic	*P*-value
	Mild (*n* = 172)	Severe (*n* = 27)			Mild (*n* = 176)	Severe (*n* = 23)		
Male [n (%)]	86 (50.0)	19 (70.4)	3.885	0.049	91 (51.7)	14 (60.9)	0.686	0.408
Age [M (IQR), year]	62.0 (18.8)	71.0 (13.0)	−3.096^b^	0.002	64.0 (16.8)	54.0 (21.0)	−1.955^b^	0.051
Hypertension [n (%)]	95 (55.2)	18 (66.7)	1.234	0.265	102 (58.0)	11 (47.8)	0.850	0.356
Diabetes mellitus [n (%)]	36 (20.9)	7 (25.9)	0.344	0.558	38 (21.6)	8 (34.8)	1.992	0.158
Smoke [n (%)]	34 (19.8)	6 (22.2)	0.088	0.767	39 (22.2)	7 (30.4)	0.784	0.376
Drinking [n (%)]	18 (10.5)	4 (14.8)	0.116	0.734	20 (11.4)	2 (8.7)	0.001	0.976
**Laboratory test**								
TG (*x* ± s, mmol/L)	4.11 ± 1.06	4.06 ± 1.18	0.217^a^	0.828	4.13 ± 1.04	3.95 ± 1.28	0.732^a^	0.465
TC [M (IQR), mmol/L]	1.11 (0.68)	0.92 (0.94)	−0.809^b^	0.419	1.12 (0.71)	1.04 (0.80)	−1.022^b^	0.307
HDL [M (IQR), mmol/L]	1.28 (0.46)	1.23 (0.36)	−0.018^b^	0.986	1.29 (0.45)	1.15 (0.38)	−1.232^b^	0.218
LDL [M (IQR), mmol/L]	2.40 (1.32)	2.55 (1.33)	−0.376^b^	0.707	2.40 (1.31)	2.50 (1.82)	−0.697^b^	0.486
Hcy [M (IQR), mmol/L]	11.7 (5.9)	13.3 (8.1)	−1.496^b^	0.135	11.6 (5.5)	14.4 (6.8)	−1.897^b^	0.058
**CSVD [n (%)]**								
sPWMH	34 (19.8)	19 (70.4)	30.582	<0.01	52 (29.5)	11 (47.8)	3.142	0.076
sDWMH	32 (18.6)	20 (74.1)	37.199	<0.01	53 (30.1)	8 (34.8)	0.209	0.648
Lacune	47 (27.3)	25 (92.6)	42.715	<0.01	66 (37.5)	12 (52.2)	1.838	0.175
CMBs	55 (32.0)	8 (29.6)	0.059	0.807	57 (32.4)	9 (39.1)	0.417	0.518
**HRV [M (IQR)]**								
SDNN (ms)	124.5 (51.3)	117.0 (44.0)	−1.702^b^	0.089	125.5 (49.5)	108.0 (49.0)	−1.671^b^	0.095
SDANN (ms)	108.0 (45.0)	106.0 (52.0)	−0.981^b^	0.326	108.0 (44.8)	105.0 (54.0)	−0.339^b^	0.735
SDNNindex (ms)	49.0 (26.8)	48.0 (32.0)	−0.124^b^	0.901	50.5 (26.8)	47.8 (38.0)	−0.763^b^	0.446
rMSSD (ms)	63.5 (50.8)	32.0 (25.0)	−6.205^b^	<0.01	58.0 (47.8)	45.0 (42.0)	−1.398^b^	0.162
PNN50 (%)	6.0 (13.0)	5.0 (7.0)	−2.158^b^	0.031	6.0 (11.0)	5.0 (19.0)	−0.559^b^	0.576
TP (ms^2^)	1,830.2 (1,819.1)	1,678.3 (772.7)	−1.837^b^	0.066	1,819.6 (1,723.3)	1,710.6 (828.0)	−1.170^b^	0.242
LF (ms^2^)	296.6 (353.5)	258.6 (224.7)	−1.571^b^	0.116	294.8 (343.0)	231.5 (193.5)	−0.747^b^	0.455
HF (ms^2^)	131.6 (167.8)	126.5 (160.6)	−1.215^b^	0.224	134.9 (171.7)	99.0 (100.2)	−1.401^b^	0.161
LF/HF	1.9 (1.0)	3.8 (4.7)	−5.133^b^	<0.01	2.0 (1.1)	2.1 (1.2)	−0.279^b^	0.780

EPVS, enlarged perivascular spaces; BG, basal ganglia; CS, centrum semiovale; IQR, interquartile range; SD, standard deviation; TC, total cholesterol; TG, triglyceride; LDL, low-density lipoprotein cholesterol; HDL, high-density lipoprotein cholesterol; Hcy, homocysteine; CSVD, cerebral small vessel disease; sPWMH, severe paraventricular white matter hyperintensity; sDWMH, severe deep white matter hyperintensity; CMBs, cerebral microbleeds; HRV, heart rate variability; TP, total power; LF, low-frequency power; HF, high-frequency power; LF/HF, the ratio of LF to HF; SDNN, the SD of normal-normal (NN) intervals; SDANN, the SD of the mean of NN intervals every 5 min for the period of interest; SDNNindex, the mean of NN intervals SD every 5 min for period of interest; rMSSD, the root mean square of successive differences of NN intervals for period of interest; PNN50, the percentage of adjacent NN intervals greater than 50 ms. ^a^*t*-value, ^b^Z-value, the residual test statistic was the *x*^2^-value.

The HRV variables that statistically differed in the univariate comparison between the BG-EPVS subgroups were analyzed *via* logistic regression, and the results after correcting for age and sex showed that rMSSD and LF/HF were factors influencing the severity of BG-EPVS (rMSSD: OR 0.909, 95% CI 0.859–0.962, *P* = 0.001; LF/HF: OR 3.893, 95% CI 1.803–8.404, *P* = 0.001). LF/HF and rMSSD remained independent influences on BG-EPVS load, after correcting for age, sex, underlying medical history, smoking history, drinking history, WMH, lacune, and CMBs (rMSSD: OR 0.871, 95% CI 0.768–0.988, *P* = 0.032; LF/HF: OR 3.854, 95% CI 1.196–12.419, *P* = 0.024) (Table 2).

**TABLE 2:** Heart rate variability (HRV) in relation to BG-EPVS load (binary logistic regression).

	*B*	*SE*	Wald *x*^2^	*P*-value	OR	95% CI
**Model 1**						
rMSSD	−0.095	0.029	10.920	0.001	0.909	0.859–0.962
PNN50	0.041	0.055	0.574	0.449	1.042	0.937–1.160
LF/HF	1.359	0.393	11.984	0.001	3.893	1.803–8.404
**Model 2**						
rMSSD	−0.138	0.064	4.600	0.032	0.871	0.768–0.988
PNN50	0.004	0.095	0.002	0.965	1.004	0.833–1.211
LF/HF	1.349	0.597	5.108	0.024	3.854	1.196–12.419

Mild BG-EPVS as the reference group. Model 1, adjusted for age and sex; Model 2, adjusted for age, sex, hypertension, diabetes mellitus, smoke, drinking, WMH, lacune, and CMBs.

### Factors influencing cognitive function in enlarged perivascular space patients

#### General information

Compared to the normal cognitive function group, the cognitive impairment group significantly was older and had a higher rate of diabetes, lower educational levels, as well as higher rates of sPWMH, sDWMH, and lacune (*P* < 0.05). No statistically significant differences were observed between the remaining groups (*P* > 0.05) (Table 3).

**TABLE 3:** Univariate analysis of factors related to cognitive impairment in enlarged perivascular space (EPVS) patients.

Characteristics	Cognitive impairment		Test statistic	*P*-value
	No (*n* = 157)	Yes (*n* = 42)		
Male [n (%)]	79 (50.3)	26 (61.9)	1.785^c^	0.182
Age [M (IQR), year]	61.0 (19.0)	72.0 (13.5)	−5.322	<0.01
Hypertension [n (%)]	87 (55.4)	30 (71.4)	3.508^c^	0.061
Diabetes mellitus [n (%)]	31 (19.7)	15 (35.7)	4.755^c^	0.029
Smoke [n (%)]	35 (22.3)	11 (26.2)	0.283^c^	0.595
Drinking [n (%)]	17 (10.8)	5 (11.9)	0^c^	1
Years of education [M (IQR), year]	9.0 (6.0)	5.5 (5.7)	−5.303	<0.01
**CSVD [n (%)]**				
sPWMH	43 (27.4)	20 (47.6)	6.268^c^	0.012
sDWMH	41 (26.1)	20 (47.6)	7.209^c^	0.007
Lacune	53 (33.8)	25 (59.5)	9.230^c^	0.002
CMBs	53 (33.8)	13 (31.0)	0.118^c^	0.732
**HRV [M (IQR)]**				
SDNN (ms)	128.0 (51.0)	117.0 (44.8)	−2.441	0.015
SDANN (ms)	109.0 (44.5)	105.0 (42.3)	−1.168	0.243
SDNNindex (ms)	49.0 (25.5)	47.0 (25.3)	−1.566	0.117
rMSSD (ms)	64.0 (52.5)	34.5 (29.5)	−5.875	<0.01
PNN50 (%)	7.0 (14.0)	3.0 (5.5)	−3.370	0.001
TP (ms^2^)	1,834.1 (1,728.6)	1,677.6 (1,278.2)	−2.513	0.012
LF (ms^2^)	312.0 (348.8)	234.8 (217.1)	−3.104	0.002
HF (ms^2^)	131.7 (177.2)	122.2 (139.6)	−1.754	0.079
LF/HF	1.9 (1.2)	2.2 (1.8)	−3.062	0.002

^c^*x*^2^-value, the residual test statistic was the *Z*-value.

#### Association between heart rate variability and cognitive function in enlarged perivascular space patients

Patients with cognitive impairment had lower SDNN, rMSSD, PNN50, TP, LF, and higher LF/HF compared to those of patients without cognitive impairment (*P* < 0.05), and the differences between the two groups in SDANN, SDNNindex, and HF were not statistically significant (*P* > 0.05) (Table 3).

**FIGURE 3:**
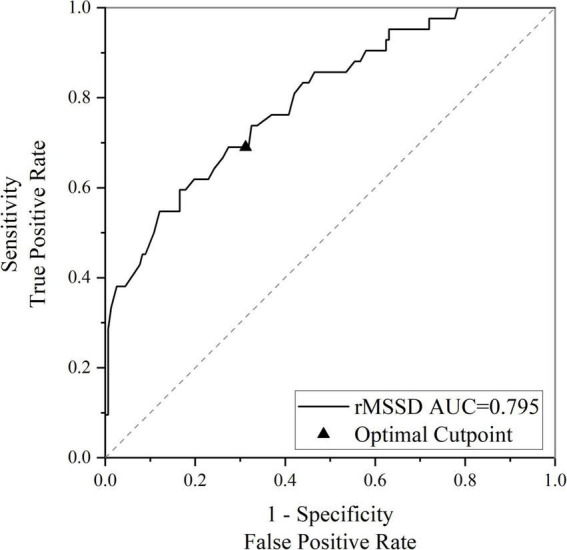
Receiver operating characteristic (ROC) curve for rMSSD predicting cognitive impairment in enlarged perivascular space (EPVS) patients. rMSSD, the root mean square of successive differences of normal-normal intervals for period of interest; AUC, area under the curve.

The HRV indicators that differed between groups in the univariate analysis were determined with logistic regression. rMSSD (OR 0.941, 95% CI 0.907–0.976, *P* = 0.001) was found to be an influential factor in cognitive impairment in EPVS patients, after correcting for age, sex, history of underlying medical conditions, smoking history, drinking history, and years of education. After further correcting for WMH, lacune, and CMBs, the time domain parameter rMSSD (OR 0.936, 95% CI 0.898–0.976, *P* = 0.002) remained an influential factor for cognitive impairment in patients with EPVS (Table 4). The rMSSD was used to build a predictive model for cognitive impairment in patients with EPVS, and the receiver operating characteristic curve was plotted with an optimal cut-off point of 0.312, area under the curve of 0.795 (95% CI: 0.719–0.872, *P* < 0.01), sensitivity of 67%, and specificity of 69% (Figure 3).

**TABLE 4:** Heart rate variability (HRV) in relation to cognitive impairment in patients with enlarged perivascular space (EPVS) (binary logistic regression).

	*B*	*SE*	Wald *x*^2^	*P*-value	OR	95% CI
**Model 1**						
SDNN	−0.012	0.014	0.784	0.376	0.988	0.962–1.015
rMSSD	−0.061	0.019	10.568	0.001	0.941	0.907–0.976
PNN50	−0.092	0.048	3.630	0.057	0.912	0.830–1.003
TP	0.001	0	2.327	0.127	1.001	1.000–1.002
LF	−0.001	0.002	0.193	0.661	0.999	0.995–1.003
LF/HF	0.347	0.215	2.599	0.107	1.414	0.928–2.155
**Model 2**						
SDNN	−0.013	0.014	0.785	0.375	0.987	0.960–1.015
rMSSD	−0.066	0.021	9.533	0.002	0.936	0.898–0.976
PNN50	−0.093	0.049	3.591	0.058	0.911	0.827–1.003
TP	0.001	0	3.264	0.071	1.001	1.000–1.002
LF	−0.001	0.002	0.450	0.502	0.999	0.994–1.003
LF/HF	0.279	0.232	1.444	0.230	1.322	0.839–2.084

No cognitive impairment group as the reference group. Model 1, adjusted for age, sex, hypertension, diabetes mellitus, smoke, and drinking; Model 2, adjusted for age, sex, hypertension, diabetes mellitus, smoke, drinking, WMH, lacune, and CMBs.

## Discussion

Enlarged perivascular space is an early imaging marker of CSVD and is a marker of brain aging that may cause a variety of adverse events, such as cognitive impairment (Groeschel et al., 2006; Wardlaw et al., 2013b). However, the underlying mechanisms associated with its actions remain largely unknown. We analyzed HRV data obtained from long-term ambulatory ECG monitoring in relation to BG-EPVS severity and its relationship with cognitive impairment in patients with EPVS. rMSSD and LF/HF independently influenced BG-EPVS severity, and rMSSD independently influenced cognitive impairment in patients with EPVS. Among the HRV parameters, rMSSD mainly reflected parasympathetic activity and LF/HF reflected sympathetic-parasympathetic balance. A decrease in rMSSD suggests a decrease in parasympathetic nervous system activity (Tarvainen et al., 2014). Therefore, the results of our study suggest that decreased parasympathetic activity and the resulting ANS imbalance may influence the development of BG-EPVS and may be a bridge to the development of cognitive impairment in patients with EPVS.

Our findings are similar to those of previous studies. Moon et al. (2018) explored the relationship between HRV and CSVD in patients with obstructive sleep apnea and found a significant correlation between decreased HRV and the development and progression of CSVD. Another two-sample Mendelian randomization analysis study reported that decreased HRV was associated with an increased risk of CSVD (Tian et al., 2021). Frewen et al. (2013) found that reduced HRV was significantly associated with reduced cognitive function in an elderly Irish population. In a cross-sectional study of the relationship between HRV and cognitive impairment in 311 community-dwelling older women, Kim et al. (2006) showed that ANS dysfunction, which particularly reduced the parasympathetic activity, was independently associated with cognitive impairment in community-dwelling older women. These findings further reinforce the theoretical aspects of our study. However, a study by Yamaguchi et al. (2015) based on community dwellers and measuring nocturnal HRV by ambulatory blood pressure showed that elevated HRV was a risk factor for CSVD progression in older adults, whereas another prospective study that included a middle-aged population under stable employment and collected 5-min HRV data exhibited no longitudinal association between HRV and cognitive decline (Britton et al., 2008). This differs from the findings of the present study, which may be related to the different methods or time periods used for HRV testing.

The mechanisms linking ANS dysfunction, especially the reduced parasympathetic activity, to the development of EPVS and its cognitive impairment have not yet been elucidated, There are several possible reasons for it: (1) ANS regulates cardiac output and the diameter of capillaries in the brain (Peppiatt et al., 2006; Krueger and Bechmann, 2010) to maintain a smooth cerebral blood flow; when it is disrupted, cerebral vascular self-regulation is impaired, which may cause hypoperfusion of the whole brain. Moreover, the lack of structural anastomosis in the BG vasculature is more likely to cause localized brain tissue ischemia and hypoxia, which subsequently triggers oxidative stress and activates an inflammatory cascade response, resulting in the disruption of the blood-brain barrier integrity, damage to glial cells and neurovascular units, and destruction of cognitively relevant nerve fibers (Chen et al., 2015). Furthermore, metabolic waste products and small microproteins flow into the perivascular space when the vascular endothelium is disrupted and, if not removed promptly, may promote the formation of EPVS. This is also a key pathogenic process of CSVD (Li et al., 2017). A recent study showed that BG-EPVS, but not CS-EPVS, was associated with impaired blood-brain barrier integrity (Wang et al., 2021), which may explain the association between reduced HRV and BG-EPVS but not CS-EPVS. An increasing number of studies have found that HRV may be associated with the development of CSVD, and the cumulative brain damage is often accompanied by more severe cognitive impairment. Therefore, we speculated that EPVS, a feature of CSVD, may be an important marker of the association between reduced HRV and impaired cognitive function. Specifically, reduced HRV may cause more severe EPVS and subsequently increase the risk of cognitive impairment in patients with EPVS. (2) Reduced parasympathetic activity leads to reduced activity of the cholinergic anti-inflammatory system in the central nervous system (Parada et al., 2013; Revathikumar et al., 2016), and upregulated inflammatory factors *in vivo* disrupt the vascular endothelium, leading to neurovascular unit damage and blood-brain barrier abnormalities (Abbott et al., 2006; Li et al., 2018). The cholinergic anti-inflammatory pathway has been extensively studied for its neuroprotective effects that control the inflammatory response (Egea et al., 2015; Kalkman and Feuerbach, 2016). Efferent vagal activity inhibits the release of pro-inflammatory cytokines and protects the organism from inflammatory responses. Parasympathetic nerve-mediated reductions in HRV are associated with elevated levels of inflammatory factors such as interleukin-6 (Sloan et al., 2007), and inflammatory factors are associated with reduced cognitive function (Schram et al., 2007; Trollor et al., 2012). Consequently, the inflammation caused by reduced HRV may mediate the relationship between HRV and both EPVS and cognitive function in patients with EPVS.

Our study was a single-center study based on a hospital population, and selection bias cannot be ruled out; therefore, caution should be exercised when extrapolating our findings to community groups. Several observational studies have reported that cerebrovascular lesions may directly contribute to ANS dysfunction (Dorrance and Fink, 2015; Constantinescu et al., 2019). HRV is impacted by motion, stress, and day-night rhythm. Although we have used longer recording epochs and tried to avoid events that may cause emotion swings in patients during HRV monitoring, we cannot eliminate the effect of emotions on HRV. Furthermore, HRV monitoring was performed during hospitalization and may not reflect daily life HRV. Future studies should exclude patients with psychiatric symptoms (anxiety, depression, etc.) or those taking psychotropic drugs, analyze 24-h and nocturnal HRV separately for a more in-depth study, and enroll larger sample sizes and monitoring models adapted to conduct prospective longitudinal studies to further explore their relationship. In addition, Apolipoprotein E (ApoE) genotype is an important genetic risk factor for cognitive impairment, and is correlated with diminished physiological complexity as measured by HRV (Cheng et al., 2009; Quintino-Santos et al., 2015). Further studies on more risk factors for cognitive impairment are needed to optimize the prediction model and better guide the clinic.

In conclusion, the decrease in rMSSD was an independent influencing factor for the aggravation of BG-EPVS load and cognitive impairment in patients with EPVS, suggesting that ANS dysfunction may be involved in the occurrence and development of BG-EPVS, and the pathophysiology of EPVS-related cognitive impairment. Our study provides theoretical evidence for exploring the early biomarkers of EPVS cognitive impairment. Dynamic ECG has been widely used in clinical practice as a non-invasive method to monitor HRV. For patients with HRV changes but without ANS symptoms, positive intervention may slow the occurrence or progression of EPVS and EPVS cognitive impairment.

## Data availability statement

The original contributions presented in this study are included in the article/supplementary material, further inquiries can be directed to the corresponding authors.

## Ethics statement

The studies involving human participants were reviewed and approved by the Ethics Committee of the Second Affiliated Hospital of Zhengzhou University (No. 2022193). The patients/participants provided their written informed consent to participate in this study.

## Author contributions

DZ, CL, YL, JC, and QL: study design. DZ, FZ, and CL: data collection. QL and CS: imaging analysis. DZ, CL, YL, and JC: interpretation of results. DZ and QL: statistical analysis and critical revision of the manuscript. HB and QL: supervised the project. All authors contributed to the article and approved the submitted version.
